# Spark Plasma Sintering of Aluminum-Based Powders Reinforced with Carbon Nanotubes: Investigation of Electrical Conductivity and Hardness Properties

**DOI:** 10.3390/ma14020373

**Published:** 2021-01-14

**Authors:** Nicolás A. Ulloa-Castillo, Oscar Martínez-Romero, Roberto Hernandez-Maya, Emmanuel Segura-Cárdenas, Alex Elías-Zúñiga

**Affiliations:** 1 Department of Mechanical Engineering and Advanced Materials, School of Engineering and Sciences, Tecnologico de Monterrey Av. Eugenio Garza Sada Sur 2501, Monterrey 64849, Nuevo León, Mexico; oscar.martinez@tec.mx (O.M.-R.); esca@tec.mx (E.S.-C.); 2 Research and Development Department, Siemens, Libramiento Arco Vial Poniente Km 4.2, Santa Catarina 66350, Nuevo León, Mexico; roberto.hernandez_maya@siemens.com

**Keywords:** multi-walled carbon nanotubes, single-walled carbon nanotubes, spark plasma sintering (SPS), electrical conductivity, aluminum powders, sample hardness

## Abstract

This paper focuses on reporting results obtained by the spark plasma sintering (SPS) consolidation and characterization of aluminum-based nanocomposites reinforced with concentrations of 0.5 wt%, 1 wt% and 2 wt% of single-walled carbon nanotubes (SWCNTs) and multi-walled carbon nanotubes (MWCNTs). Experimental characterization performed by SEM shows uniform carbon nanotube (CNT) dispersion as well as carbon clusters located in the grain boundary of the Al matrix. The structural analysis and crystallite size calculation were performed by X-ray diffraction tests, detecting the characteristic CNT diffraction peak only for the composites reinforced with MWCNTs. Furthermore, a considerable increment in the crystallite size value for those Al samples reinforced and sintered with 1 wt% of CNTs was observed. Hardness tests show an improvement in the composite surface hardness of about 11% and 18% for those samples reinforced with 2 wt% of SWNCTs and MWCNTs, respectively. Conductivity measurements show that the Al samples reinforced with 2 wt% of MWCNTs and with 0.5 wt% SWCNTs reach the highest IACS values of 50% and 34%, respectively.

## 1. Introduction

The investigation of metal matrix composites (MMCs) in recent decades has been key to improve the mechanical, electrical and thermal performances in developed composites materials with the main objective of being implemented in the industry [1,2]. In specific, aluminum-based (Al-based) nanocomposites materials have been extensively investigated due to their excellent mechanical and physical properties such as good formability, high corrosion resistance, lightweight and low melting temperature which makes them an ideal candidate for application in aerospace and automobile industries [2].

Carbon nanotubes (CNTs) have emerged as an ideal reinforcement to design novel Al-based CNT nanocomposites [3,4,5,6] due to their exceptional electrical, mechanical and thermal properties [7,8,9]. The preferred methodology for processing such nanocomposites is through powder metallurgy [6,10]. The uniform dispersion and optimal bonding of CNTs in the Al matrix have been identified as being critical to enhance its physical properties since the formation of interfacial phases between both Al and CNTs can influence mechanical, electrical and thermal properties of the Al-based CNT composite [11]. In this sense, several experimental methodologies have been addressed to achieve a uniform dispersion of nanocomponents in the preparation of such metal-based composites. For instance, solution-assisted methodologies in which the dispersion principle is based on the attachment of functional groups on both surfaces, the metal matrix and CNTs, to promote an effective interaction; see, for example, Refs. [12,13]. Moreover, the high-energy ball-milling technique has also demonstrated to be an affordable route to achieve a uniform dispersion, since it is capable to promote an effective interfacial bonding between both phases, while reducing considerable damage in the structure of the reinforcement and thus, preserving their intrinsic properties [14,15,16,17].

On the other hand, the spark plasma sintering (SPS) technique is a very useful technique of consolidation of composite powders and it is a well-known technique that produces highly densified composites, eliminates grain growth, improves the bonding in the grain boundaries, conserves energy and saves time and cost [18,19]. Several investigations have been reported previously to enhance the understanding and comprehension of Al-CNTs consolidation by varying pressure, temperature and holding time during the SPS process [19,20,21,22,23,24,25,26]. Additionally, it has been reported that faster cooling rates and short processing time during sintering avoid the formation of Al_3_C_4_ [3,14]. Efforts have been largely focused on investigating strengthening of the mechanical properties, however, there is also a scientific and industrial interest to address the electrical conductivity properties of monolithic Al-based CNT composites to use them, potentially, as replacement of conductive materials while preserving their enhanced mechanical properties. In this regard, Ujah et al. [27] recently reported that is possible to enhance not only the tribological and thermal properties, but also the electrical performance in Al-CNT composites sintered by SPS without hindering each property.

The aim of this study focuses on investigating the electrical conductivity properties of Al-based CNT nanocomposites reinforced with concentrations of 0.5 wt%, 1 wt% and 2 wt% of SWCNTs and MWCNTs to find the best experimental conditions to increase their mechanical and electrical properties. Results obtained in this study confirm that the processing of Al-based CNT nanocomposite materials by SPS is, in a first approach, an excellent candidate to manufacture specimens that can be used in electrical applications as bulk materials.

## 2. Materials and Methods

Aluminum powder (99.6% purity grade) was purchased from Jalmek, San Nicolás de los Garza, NL, Mexico. The SWCNTs (carbon basis 85%, ≥70% as carbon nanotubes with diameters between 1.3–2.3 nm) and MWCNTs (carbon basis > 90%, the diameter of the tube ranges between 110–170 nm and the length is between 5–9 μm) were purchased from Sigma Aldrich, Toluca, México. All materials were used without any further purification.

### 2.1. Dispersion and Adhesion of CNTs by High-Energy Ball Milling

The dispersion and adhesion of MWCNTs and SWCNTs in the Al metal matrix were carried out through a high-energy ball-milling machine (SPEX, 8000 Mixer/Mill High Energy Ball Mill, purchased from Advanced Analytical Systems, Guadalajara, Jalisco, Mexico). The ball-milling process was performed considering 20 grs of Al, Zircon oxide balls (10 mm diameter), ball-to-powder ratio of 10:1 and a rotation of 1200 rpm for 30 min. The descriptions of all high-energy ball-milled nanocomposite powders are listed in Table 1.

### 2.2. Spark Plasma Sintering of the Al-Based CNTs Nanocomposites

The nanocomposite powders were sintered by SPS (Dr. Sinter SPS-1050 equipment purchased from Fiju Electronic Industrial, Tsurugashima, Saitama, Japan). First, the obtained ball-milled nanocomposite powders were added into a graphite die (50 mm of diameter and 12.7 mm of wall thickness) wrapped in graphite fiber and using graphite foil as separators, which were placed on top and bottom of the system in order to avoid leakage of material. The die with the powder was placed into the vacuum chamber of the SPS equipment, and then it was compacted at 50 MPa using graphite punches. The sintering process was performed at 620 °C with a heating rate of 50 °C/min and a holding time of 5 min. The SPS equipment was operated at 1500 A and 2.5 V, the temperature of the system was monitored using a thermocouple attached to the graphite die. Subsequently, the sintered nanocomposite was released from the die once the system was cooled down to room temperature. All sintered Al-based CNT nanocomposite specimens were thermally treated in a furnace at 650 °C for 10 min in order to enhance their consolidation. The density of the sintered disk-shaped (50 mm in diameter and a thickness of 5 mm) nanocomposites samples was measured by Archimedes method using deionized water as the immersion medium.

### 2.3. Scanning Electron Microscopy (SEM)

The morphology characterization of the sintered Al-based CNT nanocomposites was carried out through a SEM (ZEISS model EVO MA 25, Oberkochen, Germany) microscope using an accelerating voltage of 20 kV and a work distance of 10 mm. The cross-sectional surface morphology characterization was investigated by analyzing secondary electrons (SE) images and the analysis of chemical composition was investigated by taking backscattered electrons (BSE) images. Additionally, energy dispersive spectroscopy (EDS) elemental mapping images were collected in order to analyze the distribution of MWCNT in samples M1 (MWCNTs at 0.5 wt%) and M3 (MWCNTs at 2 wt%).

### 2.4. X-ray Diffraction (XRD)

The structural characterization was carried out using a PanAnalytical (X’Pert Pro PW1800, Malvern, UK) system. The measurements were performed using a Bragg–Brentano geometry in reflection mode, Cu-Kα radiation in a 2*θ* range of 10°–145° with a scanning rate of 2°/min. The XRD equipment was operated at 45 mA and 40 kV. The crystallite size was calculated from the XRD patterns by applying the following modified Williamson–Hall equation [28]:

(1) $$\beta_{hkl}\cos\theta =\frac{K\lambda }{d}+4\varepsilon \sin\theta$$

where $\beta_{hkl}=\sqrt{{\left(\beta_{hkl}\right)}_{\mathrm{measured}}^{2}-{\left(\beta_{hkl}\right)}_{\mathrm{instrumental}}^{2}}$ is the instrumental broadening after incorporation of the instrumental broadening, ${\left(\beta_{hkl}\right)}_{\mathrm{measured}}$ and ${\left(\beta_{hkl}\right)}_{\mathrm{instrumental}}$ are the full width half maxima (FWHM) of the maximum intensity peak (peak position *θ*) for the experimental and standard sample (strain-free annealed pure Al sample), respectively, *K* is a constant, *λ* is the wavelength of the X-ray used, and *ε* is the induced lattice strain.

### 2.5. Microindentation Hardness Tests

The hardness surface mechanical properties of the thermally treated samples were analyzed through Hardness Vickers (HNV) tests using a Micromet (Buehler, model 5103, Lake Bluff, IL, USA) system and using an indenter made of diamond with a square-based pyramid shape (136° between faces). The tests were performed applying a force of 100 g for 15 s. The HNV values were calculated by dividing the load by the surface area of the indentation. Thereafter, the tensile and yield strengths were calculated using the method of Cahoon et al. [29], which has been used previously by Ujah et al. [30,31], given in the following equations:

(2) $$T=\left(\frac{H_{m}}{2.9}\right)\times {\left(\frac{n}{0.217}\right)}^{n},$$

(3) $$Y=\left(\frac{H_{m}}{3}\right)\times {\left(0.1\right)}^{n},$$

where T is the tensile strength of the material (MPa), Y is yield strength of the material (MPa), $H_{m}$ is microhardness of the material (N/m^2^) and *n* is the strain-hardening coefficient of the material. For this work, *n* was taken as 0.2 as reported by Callister [32].

### 2.6. Electrical Conductivity Measurements

The electrical conductivity measurements were performed using the four-wire method which setup configuration consisted of a DC system power supply (Keysight Technologies, Model 6553A, Zapopan, Jalisco, Mexico), an Agilent Technologies U3402A multimeter (Santa Clara, CA, USA) and copper wires. The electrical resistance values were measured in different lengths of the nanocomposite specimens and considering a 4 mm × 5 mm nominal cross-sectional area. The electrical resistivity of the specimens, ρ, was calculated using the equation:

(4) $$R=\frac{\rho }{A}l+2Rc,$$

where R is the resistance, Rc is the contact resistance, A is the cross-sectional area of the specimen and l is the distance between probes. The electrical resistivity was retrieved from the least-squares fitting of the data and the electrical conductivity, σ, was determined using the following equation:

(5) $$\sigma \left(S/m\right)=\frac{1}{\rho \left(\Omega \;m\right)}.$$

## 3. Results and Discussion

### 3.1. Cross-Sectional Morphology and EDS Elemental Mapping Analysis

**Figure 1:**
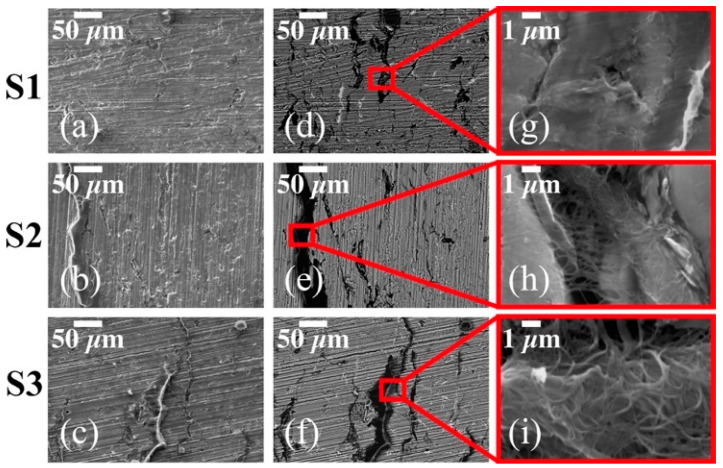
Cross-sectional SE-SEM (**a**–**c**) and BSE-SEM (**d**–**f**) micrographs for sintered nanocomposites S1, S2 and S3 reinforced with single-walled carbon nanotubes (SWCTNs) at 0.5 wt% 1 wt% and 2 wt%, respectively. The presence of the SWCNT clusters in (**g**–**i**) are caused by the strong SWCNT–SWCNT Van der Waals interaction.

The cross-sectional morphology characterization for all sintered Al-based SWCNT nanocomposites (S1, S2 and S3) is shown in Figure 1. The SE-SEM micrographs in Figure 1a–c show the presence of SWCNTs entangled together, especially in samples S2 and S3. The BSE-SEM micrographs shown in Figure 1d–f confirm this observation by additionally detecting a smaller amount of CNT clusters in sample S1. The formation of CNT clusters is caused by the strong SWCNT-SWCNT Van der Waals interaction [33] which inhibits properly the CNT deagglomeration during the ball-milling process. A close-up of such SWCNT clusters is shown in Figure 1g–i.

**Figure 2:**
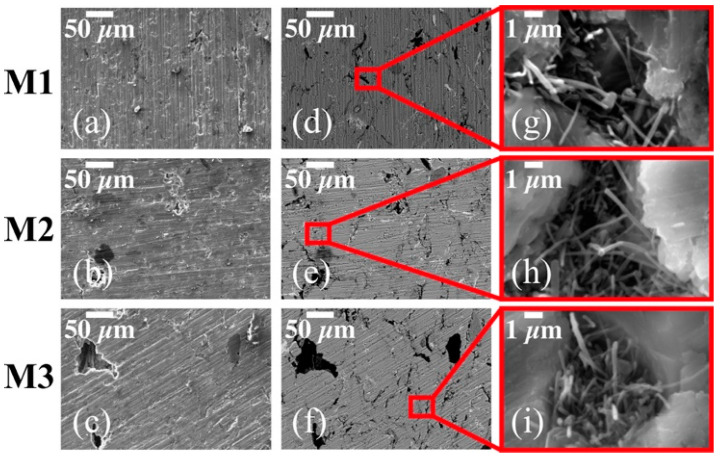
Cross-sectional SE-SEM (**a**–**c**) and BSE-SEM (**d**–**f**) micrographs for sintered nanocomposites M1, M2 and M3 reinforced with multi-walled carbon nanotubes (MWCTNs) at 0.5 wt% 1 wt% and 2 wt%, respectively. The MWCNTs agglomerations observed in (**g**–**i**) are located in the grain boundary of the Al matrix.

The corresponding SEM micrographs for sintered Al-based MWCNTs nanocomposites (M1, M2 and M3) are shown in Figure 2. The cross-sectional SE-SEM micrographs in Figure 2a–c show the presence of small micropores whose size tends to increase for higher wt% concentrations of CNT. The BSE-SEM micrographs in Figure 2d–f show the presence of small MWCNT clusters which seem to be tangled together in the grain boundary of the Al matrix. Figure 2g–i provide a close-up of such MWCNTs clusters. From the analysis performed by SEM images, it is concluded that despite the uniform dispersion achieved during the high-energy ball-milling process, it is difficult to avoid the formation of clusters due to the existing Van der Waals forces in the untreated CNTs.

**Figure 3:**
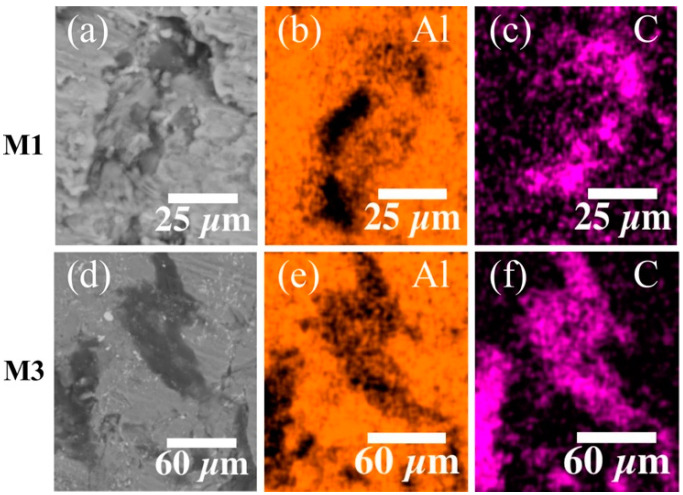
BSE-SEM micrographs (**a**,**d**) and their corresponding energy dispersive spectroscopy (EDS) elemental mapping for Al (**b**,**e**) and C (**c**,**f**) performed in samples M1 and M3. The area of MWCNTs agglomerations increases considerably at higher wt%, as observed in sample M3.

The CNT dispersion analysis was performed by EDS-SEM elemental mapping for Al and C content. The analysis was focused on those samples reinforced with MWCNTs. It was found that the best CNTs dispersion corresponds to the material samples M1 and M3. Their corresponding EDS images are shown in Figure 3. From Figure 3c, it is evident that the CNT distribution tracked for C (magenta) is relatively uniform in the Al (orange) matrix (Figure 3b). Additionally, note that the dimensions of the CNT clusters have an increment for high concentrations of CNTs, as seen in Figure 3f.

### 3.2. Densification and Crystallite Size

The effect of CNT on the relative densities measured for all Al-based CNT nanocomposites are listed in Table 2. The relative density values for samples S1 and S2 are quite similar, showing only a difference of less than 1%, while for sample S3 (2 wt% of SWCNTs) the value shows a decrement less than 2% in comparison with S1 and S2. Such behavior is attributed to the fact that sample S3 contains three times more SWCNT wt% than S1 and such amount difficult the CNT dispersion in the Al matrix since the SWCNT-SWCNT Van der Waals interaction allows the creation of large SWCNTs clusters, which are located preferably around the Al grain boundaries. This could affect the densification of the samples. Similar behavior is observed for samples reinforced with MWCNTs, in which the difference between the density values for M1–M2 is 3.4%, and for samples M1–M3 of 3.9%.

**Figure 4:**
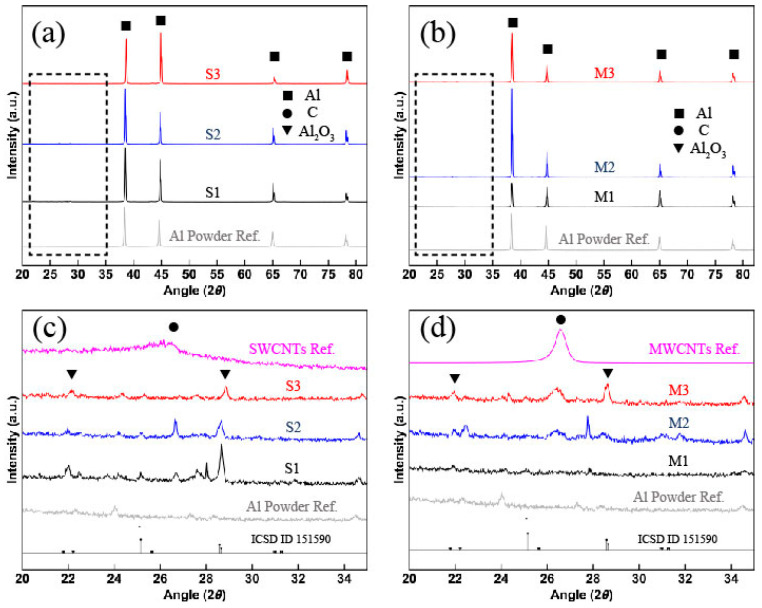
X-ray diffraction (XRD) diffractograms for sintered Al-based composites samples reinforced with (**a**) SWCNT (S1–S3) and (**b**) MWCTNs (M1–M3), showing the characteristic planes for aluminum. (**c**,**d**) are the corresponding insets of (**a**,**b**), respectively; it is possible to note the characteristic (002) plane for graphitic nanostructures detected in sintered samples. The diffractograms measured for SWCNTs, MWCNTs and Al_2_O_3_ (ICSD ID 151590) references were scaled for comparison.

The diffractograms for all sintered samples are shown in Figure 4. Note the characteristic peaks of the Al fcc (face cubic center) unit cell around 38.1° and 44.5°, which correspond to the (111) and (200) planes, as shown in Figure 4a,b. The characteristic XRD peak for graphitic structures like CNTs is commonly detected around 27° and corresponds to the (002) plane (interplanar spacing of 0.34 nm). As shown in Figure 4c, the absence of the CNT is due to the weak signal of SWCNTs as well as the fact that they were uniformly dispersed in the Al matrix. Moreover, the MWCNT signal detected for samples M1–M3 in Figure 4d shows a clear tendency of increasing its amplitude when the content of MWCNT is increased. The corresponding experimental diffractograms for SWCNTs and MWCNTs were included and scaled for a better comprehension of the results. The small peaks detected around 29° in both set of samples are attributed to Al_2_O_3_ (ICSD ID 151590) which could be formed during the sintering consolidation process.

The crystallite size computed from the XRD diffractograms by using the Williamson–Hall equation are listed in Table 2. The crystallite size for samples reinforced with SWCNTs increased considerably from 110 nm (S1) to 227 nm (S2) upon increasing from 0.5 wt% to 1 wt% the CNT concentration Similar result is observed in the calculation of the crystallite size values for material samples M1 and M2 obtaining an increment from 63 nm to 187 nm, respectively.

The decrement in the crystallite size values for nanocomposite samples S3 (114 nm) and M3 (71 nm) is mainly due to the increment of CNT clusters and micropores which hinders grain growth. The increment in the crystallite size values occurs because of the formation of a 2D CNT channel network on the surface of the Al matrix surface during ball milling, which restricts the radial plastic flow of Al crystallites. Cold welding of particles can, however, continue along the vertical direction and hence, increasing the crystallite size by increasing the CNT content [14,34]. Additionally, the heating rate used as well as the high pressure during SPS consolidation resulted in an enhanced rate of surface diffusion, accelerating the grain growth. In specific, the increased crystallite size values reported for SWCNT nanocomposite samples can be explained by the uniform dispersion of carbon nanotubes achieved during the milling process, in spite of having relatively large carbon clusters, as shown in Figure 3, the dispersion and sintering conditions were ideal to favor grain growth.

### 3.3. Hardness Tests

Table 3 shows the collected data obtained from micro-indentation hardness tests for all thermally treated Al-based CNT nanocomposite samples. As expected, the hardness, tensile and yield strength, in both set of sintered nanocomposite samples, increase for higher wt% concentrations of CNTs, indicating that the sample surface mechanical properties are strongly influenced by the CNT content and its dispersion in the Al matrix. This is confirmed from the SEM images of Figure 1 in which the SWCNT distribution for samples S1–S3 indicates that the hardness property could be affected by the presence of the CNT clusters produced during the milling process. Similarly, the hardness values obtained for samples M1–M3 confirm that when the CNT clusters are located in the grain boundaries, as illustrated in Figure 2, produce an effective mechanical reinforcement that increases the hardness of the thermally treated sample. This is consistent with the results obtained by Ujah et al. in [31] which is attributed to the homogenous dispersion of the CNTs reinforcement.

### 3.4. Electrical Conductivity Tests

**Figure 5:**
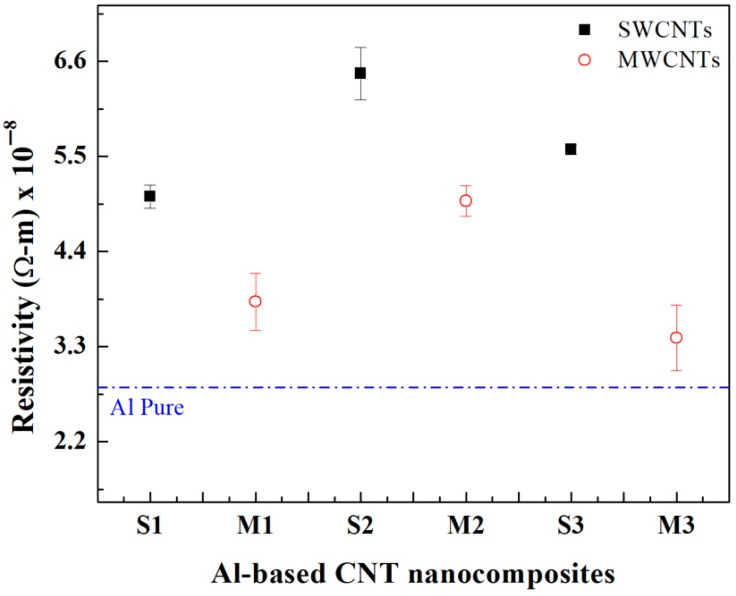
Electrical resistivity values retrieved from for Al-based nanocomposites reinforced with SWCNTs and MWCNTs. All values are compared with the standardized electrical resistivity value of pure aluminum (dashed blue line).

The sample electrical resistivity values shown in Figure 5 were measured using the four-point probe methodology [27]. The resistivity value measured for samples S1 (5.03 ± 0.13 × 10^−8^ Ω-m) is lower than those measured for samples S2 (6.45 ± 0.30 × 10^−8^ Ω-m) and S3 (5.57 ± 0.05 × 10^−8^ Ω-m) which implies that the electrical conductivity is higher for sample S1 (1.98 × 10^7^ S/m) than for samples S2 (1.54 × 10^7^ S/m) and S3 (1.79 × 10^7^ S/m). A different behavior was observed from the sintered MWCNTs nanocomposite samples, where the electrical resistivity for sample M3 (3.4 ± 0.38 × 10^−8^ Ω-m) was lower than for the values measured for samples M1 (3.82 ± 0.32 × 10^−8^ Ω-m) and M2 (4.98 ± 0.17 × 10^−8^ Ω-m). In this case, that the electrical conductivity for sample M3 (2.94 × 10^7^ S/m) is higher than the values measured for samples M1 (2.61 × 10^7^ S/m) and M2 (2.00 × 10^7^ S/m). Table 4 summarizes the collected electrical conductivity data values for all sintered nanocomposite samples as well as their International Annealed Copper Standard (IACS%) equivalent values.

The results indicate that the best electrical conductivity is attained in samples S1 and M3. The low electrical performance in samples S2 (26.7% of IACS%) and S3 (30.9% of IACS) (compared with the value for sample S1) is mainly caused by the presence of SWCNT cluster agglomerations observed in the cross-section SEM analysis of Figure 1. The relative increment in the electrical performance for sample S3 when compared with respect to the value of sample S2, is because the SWCNT networks formed within the Al matrix contribute to the carrier electron transport caused by percolation threshold phenomena [35,36]. Based on this experimental data, it is evident that the electrical conductivity of the nanocomposite samples improves for low wt% concentrations of SWCNTs without chemical treatments in order to avoid the formation of large-sized cluster agglomerations. Note that the electrical conductivity is enhanced by having a uniform dispersion of the CNTs on the Al powders. From experimental data, it was observed that the IACS measured in samples M3 are 12% and 34.6% higher than those values recorded from samples M1 and M2, respectively. The IACS% values measured in sample M3 represent an improvement of about 28% when compared to the data collected by Ujah et al. in [27]. There, they reinforced pure Al with 8 wt% of CNTs and consolidated the composite material via spark plasma sintering. They found a maximum of 39.88% IACS which is a marginal improvement of the electrical conductivity of about 2%. However, here, it was found that by adding 2 wt% of MWCNTs into the aluminum matrix, the electrical conductivity of the composite material can be increased 3%. This enhancement in electrical conductivity is attributed to the fact that the MWCNTs are strategically located in the grain boundary acting as fillers and positively contributing to the electrical percolation threshold. 

## 4. Conclusions

The results obtained in this article show that Al-based nanocomposites reinforced with unmodified CNTs and sintered via SPS are excellent candidates to manufacture materials with enhanced electrical conductivity and mechanical properties. Despite the high-energy ball-milling process, the SWCNTs were not able to be dispersed satisfactorily due to the strong Van der Waals interaction resulting in the creation of large clusters and, hence hindering their properties. This condition was not observed for the MWCNTs since experimental characterization showed that these carbon nanotubes were successfully dispersed in the aluminum matrix during the ball-milling process.

The main results obtained in this study can be summarized as follow:

(a) Al-based nanocomposites reinforced with unmodified CNTs and consolidated via SPS are excellent candidates to manufacture materials with superior hardness and electrical conductivity properties.
(b) A mechanical improvement of hardness properties is obtained in thermally treated samples M1-M3 when the CNT clusters are located in the grain boundaries. This enhancement in hardness properties is attributed to the homogenous dispersion of the CNT reinforcement.
(c) The electrical conductivity measurements show that by adding 2 wt% of MWCNTs into the aluminum matrix, the electrical conductivity of the SPS samples is increased about 3%, which is attributed to the location of the MWCNTs clusters in the grain boundaries acting as fillers and positively contributing with the electrical percolation threshold. Finally, this research sets the experimental conditions to process Al-based nanocomposites with the aim of using them in electrical applications as bulk materials.

## Figures and Tables

**Table 1:** Description of the high-energy ball-milled Al-based carbon nanotube (CNT) nanocomposite powders.

Sample	Description	Sample	Description
S1	Al + SWCNTs at 0.5 wt%	M1	Al + MWCNTs at 0.5 wt%
S2	Al + SWCNTs at 1 wt%	M2	Al + MWCNTs at 1 wt%
S3	Al + SWCNTs at 2 wt%	M3	Al + MWCNTs at 2 wt%

**Table 2:** Crystallite size and relative density of Al-based CNT nanocomposites with different wt% of CNTs.

Sample	Description	Crystallite Size (nm)	Relative Density (%)
S1	Al + SWCNTs at 0.5 wt%	110	94.3
S2	Al + SWCNTs at 1 wt%	227	94.8
S3	Al + SWCNTs at 2 wt%	114	92.6
M1	Al + MWCNTs at 0.5 wt%	63	96.2
M2	Al + MWCNTs at 1 wt%	187	92.8
M3	Al + MWCNTs at 2 wt%	71	92.3

**Table 3:** Hardness, tensile, and yield strength values of SPS thermally treated Al samples reinforced with SWNCTs (S1–S3) and MWCNTs (M1–M3) with 0.5 wt%, 1 wt%, and 2 wt%, respectively.

Sample	Hardness Vickers (MPa)	Tensile Strength (MPa)	Yield Strength (MPa)
S1	770.3 ± 13.1	261.3 ± 4.45	162.0 ± 2.76
S2	859.9 ± 12.8 (↑ 11%)	291.7 ± 4.34 (↑11%)	180.8 ± 2.69 (↑ 11%)
S3	866.4 ± 17.5 (↑11%)	293.9 ± 5.96 (↑11%)	182.2 ± 3.69 (↑ 11%)
M1	866.6 ± 1.73	294.0 ± 5.77	182.2 ± 3.58
M2	1003.0 ± 55.2 (↑ 15%)	340.2 ± 18.0 (↑ 15%)	210.9 ± 11.2 (↑ 15%)
M3	1026.8 ± 38.9 (↑ 18%)	348.3 ± 13.2 (↑ 18%)	215.9 ± 8.19 (↑ 18%)

**Table 4:** Electrical conductivity values, calculated from the electrical resistivity, and their corresponding conversion to the International Annealed Copper Standard (IACS%) values of spark plasma sintered Al-based CNT nanocomposites.

Sample	Conductivity (S/m)	IACS (%)	Sample	Conductivity (S/m)	IACS (%)
S1	1.98 × 10^7^	34.2	M1	2.61 × 10^7^	45.1
S2	1.54 × 10^7^	26.7 (↓22%)	M2	2.00 × 10^7^	34.6 (↓23%)
S3	1.79 × 10^7^	30.9 (↓9.6%)	M3	2.94 × 10^7^	50.7 (↑12%)

## Data Availability

Data available on request due to restrictions eg privacy. The data presented in this study are available on request from the corresponding author.
